# The WTC Disaster: Landrigan’s Response

**Published:** 2004-08

**Authors:** Philip J. Landrigan

**Affiliations:** Mount Sinai School of Medicine, New York, New York, E-mail: phil.landrigan@mssm.edu

My colleagues and I thank Lange for his letter confirming our finding that asbestos was present in settled dust as well as in airborne samples obtained at Ground Zero, the site of the World Trade Center, and for his having agreed with us that this asbestos almost certainly represented an exposure hazard for workers. The asbestos that was detected in the dust at Ground Zero originated from asbestos that had been sprayed onto the steel skeleton of the Twin Towers as fireproofing when the structure was being built. It was well known that asbestos was applied in the North Tower up to about the 40th story and at other locations throughout the structure before the practice of spraying on asbestos was banned in New York City in the early 1970s (Nicholson et al. 1971; Reitze et al. 1972). Concentrations of asbestos in the dust at Ground Zero were highly variable, and the level in any particular sample reflects the location of sampling and the composition of the dust that happened to be in that area. We agree with Lange’s view that workers likely had intermittent exposures to asbestos that would have arisen unpredictably when, for example, they picked up a steel beam or turned over rubble and liberated asbestos fibers into the air. The asbestos hazard to workers was magnified by the fact that the U.S. Occupational Safety and Health Administration (OSHA) failed to require constant use of respirators at Ground Zero.

We disagree strongly with Lange’s statement that “it is unlikely that exposure to asbestos itself will result in any actual health effects.” Lange appears to base his assertion, first, on the fact that most of the asbestos at the World Trade Center was chrysotile asbestos, and second, that duration of exposure for most workers was brief. Unfortunately neither of those factors conveys protection. We remain concerned that there now exists a risk for mesothelioma caused by occupational exposure to asbestos for the brave men and women who worked and volunteered at Ground Zero.

All types of asbestos fibers, chrysotile included, have been shown in laboratory as well as clinical studies to be capable of causing malignant mesothelioma (Nicholson and Landrigan 1996). All types of asbestos fibers, chrysotile included, have been declared proven human carcinogens by OSHA, the U.S. Environmental Protection Agency, and the International Agency for Research on Cancer. Pathologic studies have found short chrysotile fibers, the predominant type of fiber in World Trade Center dust, to be the predominant fiber in mesothelioma tissue (Dodson et al. 1991; LeBouffant et al. 1973; Suzuki and Yuen 2002). Moreover, mesothelioma has been reported in persons with relatively low-dose, nonoccupational exposure to asbestos of brief duration (Anderson 1982; Camus et al. 1998; Magnani et al. 2001). The greatest future risk of mesothelioma would appear to exist among first responders who were covered by the cloud of dust on 11 September 2001 as well as in other workers employed directly at Ground Zero and workers employed in cleaning asbestos-laden dust from contaminated buildings. Although we agree with Lange that the number of mesothelioma cases will probably not be great, we think it quite misleading to state that no risk exists.
